# The Association of the 24-Hour Activity Cycle Profiles With Cognition in Older Adults With Mild Cognitive Impairment: A Cross-Sectional Study

**DOI:** 10.1093/gerona/glae099

**Published:** 2024-04-20

**Authors:** Guilherme Moraes Balbim, Ryan S Falck, Nárlon Cássio Boa Sorte Silva, Arthur F Kramer, Michelle Voss, Teresa Liu-Ambrose

**Affiliations:** Djavad Mowafaghian Centre for Brain Health, University of British Columbia, Vancouver, British Columbia, Canada; Centre for Aging SMART at Vancouver Coastal Health, Vancouver Coastal Health Research Institute, Vancouver, British Columbia, Canada; Djavad Mowafaghian Centre for Brain Health, University of British Columbia, Vancouver, British Columbia, Canada; Centre for Aging SMART at Vancouver Coastal Health, Vancouver Coastal Health Research Institute, Vancouver, British Columbia, Canada; Djavad Mowafaghian Centre for Brain Health, University of British Columbia, Vancouver, British Columbia, Canada; Centre for Aging SMART at Vancouver Coastal Health, Vancouver Coastal Health Research Institute, Vancouver, British Columbia, Canada; Department of Psychology, Northeastern University, Boston, Massachusetts, USA; Beckman Institute, University of Illinois at Urbana-Champaign, Urbana, Illinois, USA; Department of Psychological and Brain Sciences, University of Iowa, Iowa City, Iowa, USA; Iowa Neuroscience Institute, University of Iowa, Iowa City, Iowa, USA; Djavad Mowafaghian Centre for Brain Health, University of British Columbia, Vancouver, British Columbia, Canada; Centre for Aging SMART at Vancouver Coastal Health, Vancouver Coastal Health Research Institute, Vancouver, British Columbia, Canada; (Medical Sciences Section)

**Keywords:** Cognition, Physical activity, Sedentary behavior, Sleep

## Abstract

**Background:**

The relationship of cognition and the 24-h activity cycle (24-HAC), encompassing physical activity, sedentary behavior, and sleep, in older adults with mild cognitive impairment (MCI) remains uncertain. Distinct combinations of 24-HAC behaviors can characterize unique activity profiles and influence cognition. We aimed to characterize 24-HAC activity profiles in older adults with MCI and assess whether differences in cognition exist across profiles.

**Methods:**

We conducted a cross-sectional analysis utilizing baseline data from 3 randomized controlled trials involving 253 community-dwelling older adults (55 + years) with MCI (no functional impairment, dementia diagnosis, and Montreal Cognitive Assessment score <26/30). Using MotionWatch8© wrist-worn actigraphy (+5 days), we captured the 24-HAC. Cognition was indexed by the Alzheimer’s Disease Assessment Scale Cognitive Plus (ADAS-Cog-Plus). Compositional data and latent profile analyses identified distinct 24-HAC activity profiles. Analysis of covariance examined whether 24-HAC activity profiles differed in cognition.

**Results:**

Four distinct activity profiles were identified. Profile 1 (“Average 24-HAC,” *n* = 103) engaged in all 24-HAC behaviors around the sample average. Profile 2 (“Active Chillers,” *n* = 70) depicted lower-than-average engagement in physical activity and higher-than-average sedentary behavior. Profile 3 (“Physical Activity Masters,” *n* = 54) were the most active and the least sedentary. Profile 4 (“Sedentary Savants,” *n* = 26) were the least active and the most sedentary. Sleep was similar across profiles. There were no significant differences in ADAS-Cog-Plus scores between 24-HAC activity profiles (*p* > .05).

**Conclusions:**

Older adults with MCI exhibited four 24-HAC activity profiles conforming to recommended physical activity and sleep guidelines. Nonetheless, cognition was similar across these profiles.

Dementia is a public health priority, with one new case detected every 3 s (1). Mild cognitive impairment (MCI) is a transitional stage between healthy cognition and dementia in which cognitive decline is greater than expected for a person’s age and educational attainment (2). MCI is further classified into two clinical phenotypes, amnestic and nonamnestic, with the subtypes of single or multiple-domain (3). Amnestic MCI single domain primarily impairs short- or long-term memory, whereas multiple-domain impairs at least one other domain (4). Nonamnestic MCI single domain is marked by impairments in attention, processing speed, language, or executive function, whereas multiple-domain refers to impairment in at least one other domain. The worldwide prevalence of MCI—regardless of its subtype—among community-dwelling adults older than 50 is 15.56% (5), with progression rates to dementia ranging from 10% to 39% in clinical settings and 3% to 21.9% in community settings (3,6,7). There is currently a lack of effective pharmacological options for treating MCI. Thus, efforts need to be directed toward understanding the role of lifestyle factors on cognition in people with MCI (8).

Physical activity (PA), sedentary behavior (SB), and sleep are three lifestyle behaviors that occupy most of the 24-h day. PA is any bodily movement produced by skeletal muscles that increases energy expenditure above resting or basal levels (9) and is classified into different intensities depending on its energy expenditure, including (1) Light PA (LPA; 1.5–2.9 metabolic equivalents [METs]) and (2) moderate-to-vigorous PA (MVPA; ≥3.0 METs) (10). SB is any waking behavior performed from a seated, reclined, or lying position that requires <1.5 METs (11). Sleep is a naturally recurring and easily reversible state characterized by reduced or absent consciousness, perceptual disengagement, and immobility, and it is typically performed while lying down (12). Notably, sleep can be characterized by both its quantity (ie, duration) and quality (eg, sleep efficiency—the proportion of each night spent sleeping while in bed trying to sleep) (13).

The interdependent relationships of PA, SB, and sleep are core components of the 24-h activity cycle (24-HAC) (12,14). Each of these behaviors is energy-independent (ie, requires different energy expenditures) and is thus mutually exclusive, with any increase in time in one behavior resulting in an equal and opposite change in at least one other behavior (13). Researchers have traditionally examined the separate associations of these behaviors with cognition, with only a few investigations examining the inter-relationship of these behaviors with cognition (15). Studying all three behaviors together is still at a relatively immature research stage but is critical for understanding how the 24-HAC affects cognition.

The current evidence examining the association of the 24-HAC with cognition in older adults with MCI has explored two key aspects: (1) how each 24-HAC behavior is separately associated with the risk of developing MCI (16–20); and (2) the relationship of each 24-HAC behavior with cognition based on MCI status (17,21,22). These investigations have revealed three key findings. First, greater PA, lower SB, and sufficient sleep quantity (6–8 h/day) and quality (eg, greater sleep efficiency) are each independently associated with better cognition and a lower likelihood of cognitive impairment (16–20). Second, individuals with MCI engage in less PA and more SB and have poorer sleep quantity and quality than their peers without MCI (17,21,22). Third, decreased odds of subsequent dementia among individuals with MCI who spend more time in PA (Hazard Ratio from 0.82 95% CI 0.79–0.86 to 0.89, 95% CI 0.85–0.93)(23), increased odds of MCI (Odds Ratio 1.56; 95% CI 1.27–1.91) (24) and dementia (Risk Ratio 1.30, 95% CI 1.12–1.51) (18) among cognitively unimpaired older adults who spend more time in SB, and increase odds of dementia (Hazard Ratio 1.40; 95:CI 1.06–1.85) among cognitively unimpaired older adults who sleep <6 h (25). Although these findings have helped elucidate how PA, SB, and sleep separately affect cognition and risk of cognitive decline in people with MCI, they are considered outside the context of the day as a whole (eg, a night of poor sleep likely affects PA engagement). These independent analyses disregard interactive and synergistic relationships affecting health (12). Potential pitfalls of exploring 24-HAC behaviors independently include incomplete measurement, misleading recommendations, and missed opportunities for understanding how these behaviors collectively affect cognition. In this context, it is critical to understand how the 24-HAC behaviors interact to promote (or deteriorate) cognition.

Compositional data analysis is an analytic approach that aims to capture the interdependent relationships of 24-HAC behaviors with health outcomes (including cognition) (26,27). This approach considers the compositions (ie, the proportion of time) of each 24-HAC behavior as its fundamental unit of analysis. Latent profile analysis can then identify groups of individuals with distinct 24-HAC activity patterns (ie, activity profiles) (28). To the best of our knowledge, studies have yet to examine what types of 24-HAC activity profiles exist in older adults with MCI or whether these activity profiles differ in cognition. Understanding whether people with MCI display different 24-HAC activity profiles and whether these profiles are associated with varying levels of cognition could help to identify people with MCI who are more at risk for cognitive decline and dementia. Thus, in community-dwelling older adults with MCI, we aimed to (1) characterize distinct 24-HAC activity profiles, and (2) determine if there are differences in cognition between 24-HAC activity profiles.

## Method

### Study Design

This was a cross-sectional analysis of baseline data collected from 262 older adults enrolled across three randomized controlled trials (RCTs) in our laboratory: (1) 96 from a study investigating the effects of a multidomain lifestyle program on sleep and cognition in older adults with MCI and poor subjective sleep (29), (2) 102 from an exercise trial designed to examine the impact of aerobic and resistance training exercises on cognition in older adults with MCI (30), and (3) 64 from an exercise trial to assess the impact of resistance training on cognition and white matter hyperintensities in older adults with MCI and with neuroimaging evidence of cerebral small vessel disease (31) (Supplementary Figure 1). The RCTs were conducted at the University of British Columbia with recruitment and data collection from November 2016 to the present. Ethical approval was obtained from the University of British Columbia’s Clinical Research Ethics Board (H16-01029, H15-02181, H15-00972) and Vancouver Coastal Health Research Institute (V16-01029, V15-02181, V15-00972). Participants of the 3 RCTs provided written informed consent per the Declaration of Helsinki before study enrollment.

### Participants

Two hundred and sixty two community-dwelling older adults with a research-based diagnosis of amnestic or nonamnestic MCI, defined as no functional impairment, no diagnosis of dementia, and a Montreal Cognitive Assessment (MoCA) score <26/30, participated in the study. The absence of functional impairment was operationalized based on shared criteria of the included studies, specifically that participants were: (1) in sufficient health to participate in regular PA; (2) able to walk independently (with or without an assistive device); and (3) living independently in their own homes (ie, not residing in assisted-living or long-term care homes). Each RCT’s specific inclusion and exclusion criteria are published elsewhere (29–31) and displayed in Supplementary Table 1. Briefly, we included participants (1) aged ≥55 years; (2) living independently in their homes; (3) with MCI as determined by a MoCA score of <26/30 (32); 4) who scored >20/30 on the Mini-Mental State Examination (MMSE) (33); and (5) provided informed consent. We excluded those who (1) had a formal diagnosis of neurodegenerative disease, stroke, dementia (any type), or psychiatric condition; (2) were taking psychotropic medication; (3) were living in a nursing home, extended care facility, or assisted-care facility; and (4) were planning to participate or already enrolled in a clinical drug trial or exercise trial concurrent to the RCTs. The MoCA cutoff score <26/30 is a widely adopted cutoff due to its high specificity in correctly identifying MCI. In a large sample of MCI individuals (32), a MoCA cutoff score <26/30 correctly identified 90% of the individuals clinically diagnosed with MCI. In addition, the MMSE score of ≥20/30 was adopted to capture individuals with MCI but not dementia (34). MMSE scores 21–25/30 correspond to a Clinical Dementia Rating (CDR) of 1.0 (ie, mild cognitive impairment) (35). Across the three studies, the lowest MMSE cutoff was 21 (or >20/30) for inclusion (Supplementary Table 1). Participants’ MoCA scores ranged from 11 to 26 and MMSE scores ranged from 21 to 30. The RCTs’ inclusion criteria had some differences. For example, Falck and colleagues (29) included those with poor subjective sleep quality (score >5/21 on the Pittsburgh Sleep Quality Index (36)), Liu-Ambrose and colleagues (31) included those who fulfilled clinical criteria for cerebral small vessel disease (37), and Barha and colleagues (30) included those with CDR (38) <1 (Supplementary Table 1). We added RCT as a covariate in our inferential statistical analysis.

### Measurements

#### Demographic information

Demographic variables included age, height (m), weight (kg), biological sex, and educational attainment. The body mass index (BMI) was calculated (kg/m^2^), and cognitive status was assessed with the MMSE and MoCA. MoCA scoring rules adjust for ≤12 years of education, adding 1 point to the total score of individuals with ≤12 years of education.

#### Cognition

We measured global cognition with the Alzheimer’s Disease Assessment Scale—Cognitive Plus (ADAS-Cog-Plus) (39). The ADAS-Cog-Plus (39) combines executive functions and verbal fluency tasks with the 13-item ADAS-Cog score (40).We included the Trail Making Test parts A and B (41), digit span forward and backward (42), and category fluency (vegetables and animals) (43). Lower ADAS-Cog-Plus scores indicate better global cognition. According to the Alzheimer’s Disease Neuroimaging Initiative (ADNI) sample (44), cognitively healthy older adults have a mean ADAS-Cog-Plus score of approximately −1.0, individuals living with MCI around 0.0, and those living with dementia around 1.0.

#### 24-h activity cycle behavioral measurement

We measured PA, SB, and sleep duration using the MotionWatch8© (MW8; CamNtech, Cambridge, UK), a tri-axial accelerometer designed to observe acceleration ranging in magnitude from 0.01G to 8G, with a frequency of 3–11 Hz. The MW8 is a noninvasive, lightweight, battery-powered wrist-worn device with a more comfortable placement for sleeping and daytime wear (45,46). The device has evidence for measuring older adult PA and SB (46) against indirect calorimetry, wherein the classification accuracy of SB and PA was moderately accurate (46). Participants continuously wore the MW8 on their nondominant wrist for seven consecutive days. Five days of wear time provides reliable estimates of PA, SB, and sleep duration for people with and without MCI (21). We only included participants with ≥5 days of consecutive PA, SB, and sleep data, resulting in an analytic sample of *N* = 253, specifically, *n* = 88 (29), *n* = 89 (30), and *n* = 76 (31) (Supplementary Figure 1).

Participants were fitted with the MW8 for each study and provided detailed information on its features (ie, light sensor, event marker button, and status indicator). Participants were instructed to press the event marker button each night when they started trying to sleep and again each morning when they finished trying to sleep. The MW8 is the updated version of the Actiwatch 7, a device with evidence of validity against polysomnography in healthy adults (47). We used an automated rest-interval scoring algorithm of illuminance and motion data to classify time spent sleeping each night versus time spent awake each day; this algorithm has a strong correlation (*r* = 0.92) with the traditional method for determining sleep duration (48). Naps were not included in our calculations for sleep duration and were indexed as SB. Our estimates only included the major sleep period. The MW8 algorithm uses a function to categorize sleep time as consecutive epochs of <20 counts/min. Instances wherein activity exceeded 20 counts/min were scored as time awake (48). Hence, wake after sleep onset (WASO) was not included in estimates of sleep duration. These minutes wherein the participant was classified as awake were excluded from daily sleep calculations. For time classified as awake, MVPA, LPA, and SB were classified using established cutpoints for MW8 (46). Specifically, MVPA was classified as ≥562.50 counts/min, LPA as 178.51 to 562.49 counts per minute, and SB as ≤178.50 counts/min (46).

#### Determination of 24-h activity cycle activity profiles

The 24-HAC behaviors data were analyzed using Motionware 1.0.27 (CamNtech) actigraphy. Data prior to recorded wake-time on the first full day of recording were manually removed in order only to investigate complete 24-h recordings of activity. Each day of PA and SB consisted of a time when the participant was assumed to be awake and out of bed, based on the rest–wake interval scoring algorithm (48).

We deemed each 24-HAC as the time between when the participant went to bed for a given night and the last minute of recorded PA or SB the next day. For example, if a participant went to bed at 10:00 pm, woke up at 6:00 am, and went to bed at 11:00 pm, we assumed that the 24-HAC referred to the window between 10:00 pm and 10:59 pm of the next day. It is important to note that although we call each “day” of data the 24-HAC, each “day” does not refer to precisely 24 h. In the example, the participant had a “day,” which lasted 24 h and 59 min. We then averaged the time spent in each 24-HAC behavior and the day’s total duration (ie, the sum of all 24-HAC behaviors) across the recording period of >5 days.

Compositional data analysis requires a constant-sum constraint on the sample values (49), for example, 1 440 min for a day. To account for that, we double-checked that the averaged sum of each 24-HAC had the same finite scale (ie, 1 440 min). We rescaled the participants’ time spent in each 24-HAC behavior, to sum up to 1 440 min, using the closure function from the *compositions* R package (version 2.0–4). Next, we transformed the averaged rescaled time spent on each behavior into a proportion (%). For example, if a participant spent an average of 240 min engaging LPA on their 1 440 min days of recording, then the estimated % LPA for that participant would be:


$$\begin{array}{l} \frac{240\;min\;of\;LPA}{1\;440\;min\;of\;the\;day}\times 100=17.14\%\;of\;days\;spent\;in\;LPA \end{array}$$

Compositional data analysis techniques address limitations caused by multicollinearity due to the inclusion of all 24-HAC behaviors within a single model (26). It involves expressing time spent (or proportion of time) engaging in different time-use behaviors during a finite period (eg, 1 440 min or 100%) in relative terms as a set of isometric log-ratio coordinates (28). These coordinates contain all the relative information regarding the 24-HAC time-use behaviors. They can be used as vectors in standard statistical models instead of raw min/day or proportion of the day vectors.

Using the *compositions* package (version 2.0–4), we created the isometric log-ratio coordinates using a sequential binary partition process (50). To compute the first pivot coordinate, we coded % of time spent in one behavior as the numerator (+1) in the equation and % of time amongst remaining behaviors as the denominator (−1). The first behavior was partitioned out and coded as an uninvolved part (0) in the sequential binary partition to create the second coordinate. The percentage of time spent engaging in one of the remaining three behaviors was coded in the numerator (+1) and the remaining two in the denominator (−1). Next, the behavior in the numerator was partitioned out to code the last coordinate. The percentage of time engaging in the remaining two behaviors was coded in the numerator (+1) and the denominator (−1). The isometric log-ratio transforms each behavior’s composition to a unique behavior-1 vector on a new coordinate system where each new coordinate is a log-ratio that falls along the real line (28). We chose the following coordinates (1) the first coordinate included all relative information regarding MVPA versus the geometric mean of % of time spent engaging in SB, light PA, and sleep; (2) the second coordinate represented SB versus geometric mean of light PA and sleep; and (3) the last coordinate included light PA versus sleep. This set of isometric log-ratios was also adopted by Wu and colleagues (28). The isometric log-ratios transformations of the 24-HAC behaviors were expressed using equations (1)–(3), where the numbers preceding the log-ratios are normalizing constants necessary for the desirable mathematical properties of the transformed coordinates:


$$\begin{array}{l} ilr_{1}^{\left(MVPA\right)}=\sqrt{\frac{3}{4}}\;ln\frac{MVPA}{{\left(SB\;\times \;Light\;PA\;\times \;Sleep\right)}^{\frac{1}{3}}} \end{array}$$ (1)

$$\begin{array}{l} ilr_{2}^{\left(SB\right)}=\sqrt{\frac{2}{3}}\;ln\frac{SB}{{\left(Light\;PA\;\times \;Sleep\right)}^{\frac{1}{2}}} \end{array}$$ (2)

$$\begin{array}{l} ilr_{3}^{\left(Light\;PA\right)}=\sqrt{\frac{1}{2}}\ln\frac{Light\;PA}{Sleep} \end{array}$$ (3)

We used the three isometric log-ratio coordinates to perform latent profile analysis using the *tidyLPA* R package (version 1.1.0). The latent profile analysis is a data-driven approach to deriving mutually exclusive profiles. This method uses finite mixture latent modeling to generate homogenous profiles within a group (ie, similar engagement in each 24-HAC behavior relative to the others) and heterogenous profiles between groups. We chose the model based on five fit statistics: (1) Akaike information criterion (AIC) and Bayesian information criterion (BIC): both are indicators of the best balance between the simplicity of the model and its goodness of fit. Lower values indicate a more parsimonious model (51); (2) entropy: indicates the precision of classifying cases into the different profiles, determining classification accuracy. Entropy values closer to 1 indicate better accuracy (52); (3) bootstrapped likelihood ratio test *p*-value (BLRT-*p*): indicates whether the model with the *k*th profile has improved model fit compared to the previous model (53); and (4) the number of participants in each profile was ≥25 or >1% (54).

### Statistical Analysis

All analyses were conducted in R (version 4.2.2) and R Studio (version 2023.06.0). The statistical code is available on GitHub (link). All participants with analyzed data had complete data. We checked all 24-HAC behaviors, isometric log-ratio coordinates, and ADAS-Cog-Plus scores for outliers outside Q1 − 1.5 × Interquartile range (IQR) and Q3 + 1.5 × IQR (55). We winsorized observations outside the lower and upper limits in instances where outliers were detected (*n* = 17 for 24-HAC behaviors, *n* = 5 for isometric log-ratio coordinates, and *n* = 4 for ADAS-Cog-Plus). We replaced below the lower limit with the value of the 5th percentile, and the values above the upper limit with the 95th percentile (56). Differences between 24-HAC activity profiles in demographic characteristics and 24-HAC behaviors were investigated using analysis of variances (ANOVA) for continuous variables and chi-square tests for categorical variables. As post hoc comparisons, significant differences in 24-HAC activity profiles were decomposed using pairwise comparisons. As a sensitivity analysis, we examined whether RCT cohorts differed in continuous or categorical variables using ANOVA and chi-square tests, and whether results withstood without the winsorization of outliers.

To determine whether ADAS-Cog-Plus performance differed by 24-HAC activity profile, we performed an analysis of covariance (ANCOVA) wherein we adjusted for age, biological sex, BMI, MoCA score, and RCT cohort. In the event of a significant one-way ANCOVA, pairwise comparisons between groups were used. Given that this was an exploratory analysis, we did not adjust for multiple comparisons.

## Results

### Participants’ Characteristics

Descriptive characteristics, time and proportion of time spent engaging in each 24-HAC behavior, total day duration, and cognition are presented in Table 1. Overall, participants had a total day duration of 1 343.53 min (*SD* = 44.11, Range: 1 177.71–1 525.00). Participants were highly educated (university degree, *n* = 136 [53.8%]), primarily female (*n* = 157 [62.1%]), with a mean age of 73.69 years (*SD* = 5.41) and BMI of 26.32 kg/m^2^ (*SD* = 4.58), and ADAS-Cog-Plus score of −0.31 (*SD* = .62). Demographic characteristics stratified by RCT are described in Supplementary Table 1. We determined that MMSE and MoCA scores were modestly higher for participants in the RCT of Falck et al. (29) (mean MoCA score: 22.51; mean MMSE score: 28.17) in comparison with the RCTs of Barha et al. (30) (mean MoCA: 21.87; mean MMSE: 27.53) and Liu-Ambrose et al. (31) (mean MoCA: 21.74; mean MMSE: 27.46).

**Table 1.** Demographic and Clinical Characteristics of Study Participants

Variables*	All Participants (*n* = 253)
Age, years	73.69 (5.41)
Females, *n* (%)	157 (62.10)
BMI, kg/m^2^	26.32 (4.58)
MoCA, score	22.05 (2.85)
MMSE, score	27.73 (1.90)
Education, *n* (%)	
High school or less	34 (13.40)
Some university	58 (22.9)
Trade school	25 (9.9)
University degree	136 (53.8)
24-hour activity cycle behaviors	
Sleep, %/day	29.80 (3.74)
Sedentary behavior, %/day	43.55 (8.23)
Light PA, %/day	19.91 (5.08)
Moderate-to-vigorous PA, %/day	6.74 (4.57)
Total day duration, min^†^	1 343.53 (44.11)
Cognition	
ADAS-Cog-Plus, score^‡^	−0.31 (0.62)

*Notes*: ADAS-Cog-Plus = Alzheimer’s Disease Assessment Scale-Cognitive-Plus; BMI = body mass index; MMSE = Mini-Mental State Examination; MoCA = Montreal Cognitive Assessment; PA = physical activity. Twenty-four-hour activity cycle behaviors are presented as compositional means, which represent the geometric average of time use values after rescaling with the closure function from *compositions* R package (closing behaviors composition to sum up to 100%).

*Data presented either as mean (standard deviation) or count (%) where applicable.

^†^Total day duration before rescaling for sum constraint of 1 440 minutes.

^‡^Lower scores represent better performance.

### Latent Profile Analysis of 24-h Activity Cycle Behaviors

**Figure 1.**
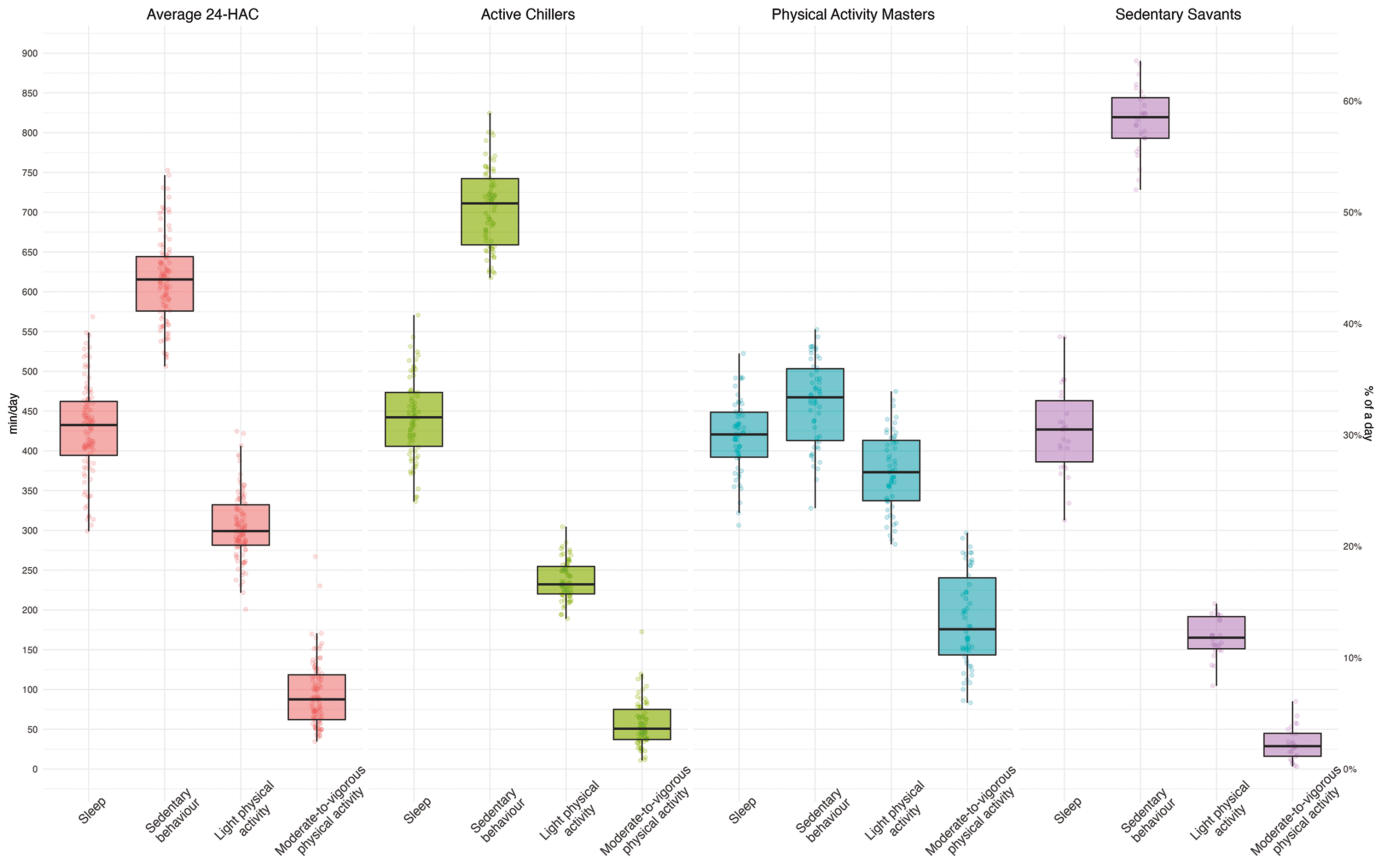
Latent profile analysis of the compositional isometric log-ratios of sleep, sedentary behavior, light physical activity, and moderate-to-vigorous physical activity by profile assignment of older adults with mild cognitive impairment.

The latent profile analysis revealed that four 24-HAC activity profiles provided the best model fit. Supplementary Table 2 depicts the results of consecutive latent profile models used to detect data-driven 24-HAC activity profiles in our sample, along with its fit statistics. Figure 1 displays the percentage of time spent in each of the four 24-HAC behaviors as a function of the latent activity profile.

Profile 1 participants (*n* = 103) had engagement similar to the overall mean in all 24-HAC behaviors. Profile 2 participants (*n* = 70) were less physically active than Profile 1, engaging in lower-than-mean engagement physical activity and higher-than-mean sedentary behavior. Henceforth we will refer to profile 1 as the “Average 24-HAC” profile and profile 2 as the “Active Chillers.” Profile 3 participants (*n* = 54) were the most active and the least sedentary. Henceforth we will refer to this profile as the “Physical Activity Masters” profile. Profile 4 participants (*n* = 26) were the least active and the most sedentary, which we will refer to as the “Sedentary Savants.” Sleep duration was similar across the 4 activity profiles.

### Differences in 24-h Activity Cycle Profiles

Table 2 describes between group differences in 24-HAC activity profiles. There was a significant difference in the proportion of females between 24-HAC activity profiles (*p *= .001) such that the proportion of females in “Average 24-HAC,” “Active Chillers,” and “Physical Activity Masters” was significantly higher compared with “Sedentary Savants” (all *p*s < .05). We also determined that 24-HAC activity profiles varied in BMI (*p* = .006), whereby participants in “Sedentary Savants” had a significantly higher BMI compared with “Physical Activity Masters” (*p* = .001). As expected, the percentage of time spent in SB, LPA, and MVPA differed between all four activity profiles (all *p*s < .001). Sleep duration did not differ between activity profiles (*p* > .05). Regarding cognition, there were no significant differences in ADAS-Cog-Plus scores between 24-HAC activity profiles (*p* = .655).

**Table 2.** Demographics, 24-HAC Behaviors, and ADAS-Cog-Plus by Profile

Variable	Overall (*n* = 253)	Average 24-HAC (*n* = 103)	Active Chillers (*n* = 70)	Physical Activity Masters (*n* = 54)	Sedentary Savants (*n* = 26)	*p*
	*M* (*SD*)	*M* (*SD*)	*M* (*SD*)	*M* (*SD*)	*M* (*SD*)	
Demographics						
Age, years	73.69 (5.41)	74.05 (5.21)	74.09 (4.97)	72.16 (5.37)	74.52 (6.90)	.116
Females, *n* (%)	157 (62.10)	71 (68.93)	39 (55.71)	40 (74.07)	7 (26.92)	<.001*
BMI, kg/m^2^	26.32 (4.58)	26.65 (4.69)	26.23 (4.12)	24.79 (4.10)	28.42 (5.35)	.006^†^
MoCA	22.05 (2.85)	22.13 (2.95)	22.19 (2.92)	21.94 (2.80)	21.62 (2.37)	.824
MMSE	27.73 (1.90)	27.97 (1.72)	27.43 (2.32)	27.74 (1.76)	27.58 (1.58)	.313
Day duration	1 343.53 (44.11)	1 341.75 (37.25)	1 349.09 (45.22)	1 327.76 (54.10)	1 368.37 (28.78)	.001^‡^
24-HAC behaviors (%/day)						
Sleep	29.80 (3.74)	29.65 (4.04)	30.64 (3.54)	29.08 (3.19)	29.62 (3.93)	.123
Sedentary behavior	43.55 (8.23)	42.65 (3.78)	48.94 (3.52)	31.99 (3.73)	56.64 (2.93)	<.001^§^
Light PA	19.91 (5.08)	21.19 (2.90)	16.48 (1.66)	25.95 (3.37)	11.55 (1.76)	<.001^§^
MVPA	6.74 (4.57)	6.51 (2.83)	3.95 (2.02)	12.99 (4.14)	2.19 (1.45)	<.001^§^
Cognitive function						
ADAS-Cog-Plus	−0.31 (0.62)	−0.25 (0.05)^‖^	−0.22 (0.06)^‖^	−0.31 (0.07)^‖^	−0.31 (0.09)^‖^	.655^‖^

*Notes*: 24-HAC = 24-hour activity cycle; ADAS-Cog-Plus = Alzheimer’s Disease Assessment Scale-Cognitive-Plus; BMI = body mass index; MMSE = Mini-Mental State Examination; MoCA = Montreal Cognitive Assessment; MVPA = moderate-to-vigorous physical activity; PA = physical activity; RCT = randomized controlled trial. Data are presented either as mean (standard deviation) or count (%) where applicable. Results are presented for analysis of variance models for continuous variables and chi-square test for categorical variables unless otherwise noted.

*p < .05 between “Average 24-HAC” versus “Sedentary Savants,” “Active Chillers” versus “Sedentary Savants,” and “Physical Activity Masters” versus “Sedentary Savants.”

^†^ *p* < .05 between “Physical Activity Masters” versus “Sedentary Savants.”

^‡^ *p* < .05 between “Average 24-HAC” versus “Sedentary Savants,” “Active Chillers” versus “Physical Activity Masters,” and “Physical Activity Masters” versus “Sedentary Savants.”

^§^ *p* < .05 between all the activity profiles.

^‖^Results presented as analysis of covariance model with data by activity profile presented as estimated marginal means and standard error, adjusted for biological sex, age, BMI, MoCA, and RCT (*F*_(9, 243)_ = 24.7).

### Sensitivity Analysis

RCT cohorts were similar across all demographic characteristics, MoCA score, 24-HAC behaviors, and ADAS-Cog-Plus score. Falck and colleagues (29) participants had a higher MMSE score than the other two RCTs (*p *= .026; Supplementary Table 3). The latent profile analysis revealed that four 24-HAC activity profiles provided the best model fit regardless of outlier winsorization (Supplementary Table 4). Results on differences between 24-HAC activity profiles withstood without outlier winsorization (Supplementary Table 5). Regarding cognition, there were no significant differences in ADAS-Cog-Plus scores between 24-HAC activity profiles without outlier winsorization (*p* = .693, Supplementary Table 5) and with education as a covariate instead of MoCA score (*p *= .929).

## Discussion

In this cross-sectional study, we identified four distinct 24-HAC activity profiles among community-dwelling older adults with MCI; each activity profile demonstrated distinct 24-HAC compositions (ie, proportions of time). However, cognition measured with ADAS-Cog-Plus did not differ between the four 24-HAC activity profiles.

These four 24-HAC activity profiles differentiated between those: (1) engaging around the overall mean in all 24-HAC behaviors (“Average 24-HAC”); (2) engaging in lower-than-mean PA and higher-than-mean SB (ie, “Active Chillers”); (3) engaging in the greatest amounts of PA and the lowest amount of SB (ie, “Physical Activity Masters”); and (4) engaging in the lowest amount of PA and the greatest amount of SB (ie, “Sedentary Savants”). Sleep duration was similar across all activity profiles. Although there is no clear evidence on optimal combinations of 24-HAC behaviors for cognition, the Canadian 24-h Movement Guidelines (57) suggest positive overall health benefits for older adults engaging in ≥150 min/week of MVPA (~1.4% of the day), several hours/day of LPA, limiting SB to 8 h/day (~33% of the day), and sleeping 7–8 h/day (~29% to 33% of the day). Importantly, we rescaled our raw estimates based on varying days’ length to sum up to a constraint of 100%. Therefore, the presented estimates are relative to the proportions of a 1 440-min day and are comparable with Canadian 24-h Movement Guidelines (57).

Our estimates of 24-HAC behaviors should be treated with caution. First, we used estimates derived from wrist-worn actigraphy, which differ from previous hip-worn actigraphy surveillance estimates (58,59) and hip- and thigh-worn estimates from compositional data analysis studies included in the Canadian 24-h Movement Guidelines (57). However, the estimates from wrist-worn actigraphy are moderately to highly correlated with hip-worn estimates (Spearman’s *r* = 0.73) (60) and demonstrate high accuracy (sensitivity >90% and specificity >95%) for classifying light PA, MVPA, and sleep compared with thigh-worn estimates (sensitivity and specificity >99%) (61). Second, our sample may be more physically active and less sedentary than the population with MCI. All participants were from British Columbia, Canada’s most physically active province, had a high educational level, and were enrolled in a lifestyle intervention. This is reflected in the fact that the smallest activity profile was the “Sedentary Savants” (*n* = 26). Last, although sleep duration did not substantially vary across the activity profiles, we observed a substantial range in sleep duration, between 227.75 and 430.86 min/night. Thus, we are unsure about how sleep duration may differ in our sample versus cognitively unimpaired or MCI populations. In a comparison of sleep profiles between cognitively unimpaired individuals, those with amnestic-MCI or with Alzheimer’s disease, there was no significant difference in MW8-assessed sleep duration between cognitively unimpaired and those with MCI (62). However, sleep durations were substantially longer in people with Alzheimer’s disease than in individuals with amnestic-MCI (62). Hence, it seems plausible that our estimates of sleep duration in people with MCI may be generalizable.

Currently, it is unclear whether cognitively unimpaired and those with MCI differ in sleep duration (62). The evidence is more clear for other aspects of sleep quality. Night-to-night variability in sleep duration and sleep fragmentation is more pronounced in people with MCI than individuals without MCI (21). People with MCI have more time awake after sleep onset than cognitively unimpaired individuals (63). Although it is unclear whether or not sleep duration differs between individuals with and without MCI, there is good evidence that sleep quality is worse in people with MCI. The relationship between sleep duration and cognition also appears to follow an inverse U-shaped relationship, wherein both long (ie, >9 h) and short sleep durations (<6 h) are negatively associated with cognitive performance (64). Sleep is a complex construct, and it remains plausible that other aspects of an individual’s sleep profile (eg, variability, architecture, efficiency, or subjective sleep quality) may better characterize individuals with MCI cognition above and beyond sleep duration.

Although our data suggests an under-representation of participants in the low PA and high SB activity profile (ie, “Sedentary Savants”), this group would be the ideal target population for interventions to improve 24-HAC composition and cognition. Although our study cannot provide direct evidence that this composition is associated with worse cognition, conceptually, this population would likely benefit the most from improvements to 24-HAC behavior compositions. This still leaves an unanswered question: does older adult cognition—for both cognitively unimpaired and impaired—differ according to 24-HAC composition?

Only a few cross-sectional studies have investigated cognition and 24-HAC composition in older adults (28,65–68), with equivocal evidence. Wu and colleagues (28) investigated whether the daily composition of sitting, standing, and stepping (no sleep data) was associated with global cognition among 1 034 cognitively unimpaired older adults (+65 years). The authors identified 4 separate activity profiles: (1) mean PA and SB; (2) above-mean PA and less SB; (3) very high PA and very low SB; and (4) very low PA and very high SB. However, global cognition did not vary according to the 24-HAC profiles. Mellow and others (65) explored the relationship between 24-HAC composition and cognition among 384 cognitively unimpaired older adults (60 + years), finding that 24-HAC composition was broadly unassociated with global cognition, memory, executive function, and processing speed. Dumuid and colleagues (67) cross-sectionally examined the association of 24-HAC compositions with cognition in 91 community-dwelling cognitively unimpaired adults (50–80 years), finding that: (1) 24-HAC composition was associated with both global cognition and executive performance; and (2) those with greater genetic risk for dementia incurred greater benefits from reallocating time to MVPA. Fanning and colleagues (66) used isotemporal substitution—a method that estimates changes in a health outcome for reallocation of time between 24-HAC behaviors—to determine that replacing SB with sleep and MVPA promoted better executive functions among 247 cognitively unimpaired older adults (60–79 years). In a sample of 3 086 older adults (60 + years) from the National Health and Nutrition Examination Survey, Wei and colleagues (68) determined that replacing 30 min of SB with 30 min of MVPA was associated with better executive function, processing speed, memory, language, and global cognition among cognitively unimpaired older adults sleeping no longer than 7 h/night while replacing 30 min of sleep with SB or MVPA was associated with better executive function, processing speed, memory, language, and global cognition among those who slept longer than 7 h/night. Differences in results may be explained by significant differences between previous studies and ours, such as the inclusion of cognitively unimpaired older adults, analysis of two instead of three 24-HAC behavior compositions (28,66,68), not applying compositional data analysis and testing predicted cognition after replacing one 24-HAC behavior with another (66,68), and assessing cognitive domains in addition to global cognition (65,66,68). Therefore, our findings add to the current literature by including older individuals with MCI and employing robust methodologies to perform compositional data analysis and derive individual profiles based on the 24-HAC.

It is thus unclear whether 24-HAC compositions are associated with cognition in people with or without MCI. Possible explanations for the inconsistency in study findings include: (1) the lack of consensus on associations between 24-HAC compositions and cognition, which precludes the recommendation of optimal 24-HAC profiles for cognition; (2) the type of analyses conducted (eg, examining group differences in cognition between 24-HAC activity profiles rather than exploring how time-reallocations are associated with cognition); (3) the characteristics of the recruited samples (15). For example, in our study, all four profiles met current PA and sleep recommendations for older adults (ie, several h/day of LPA, 150 min/week or ~1.4%/day of MVPA, and 7–8 h/night of sleep or ~29%–33% of the day), but only one activity profile (ie, “Physical Activity Masters”) met current SB guidelines of <33%/day (57); 4) bidirectionality between 24-HAC behaviors such that sleep duration affects engagement in PA and SB, and vice-versa; and (5) bidirectionality between 24-HAC compositions and cognition, in which the adequate levels of engagement in 24-HAC behaviors render similar cognition, which in turn influence subsequent activity patterns.

Sufficient PA and sleep are consistently linked to better cognition, and adequate PA may offset the negative consequences of poor sleep (69). However, the negative consequences of too much SB on cognition are far more circumspect, with systematic reviews suggesting that SB is both associated and unassociated with cognition (70). A possible reason for such inconsistent results may be that SB can be cognitively passive (eg, sitting, television watching) or active (eg, reading, playing cards) (12), and each type of SB appears to have distinct associations with cognition (65). Adding to the complexity of 24-HAC behaviors when not treated separately, PA may also ameliorate the negative consequences of SB on cognition (71). Thus, it seems plausible that our participants’ healthy PA and sleep profiles may have precluded our ability to detect differences in cognition between 24-HAC activity profiles.

Noticeably, our participants are likely people with high cognitive and brain reserve due to their high education, socioeconomic status, mostly healthy activity profiles, and low BMI, yet they live with MCI. This may mean they have significant pathologies surpassing the cognitive reserve threshold, hence manifesting cognitive impairment. In this case, it may be harder to identify the influence of activity profiles on cognition. We suggest that perhaps only extremely unbalanced activity profiles—such as a combination of very low PA, short sleep duration, and excessive SB—may further harm this population’s cognition.

The 24-HAC is a complex, dynamic, and interdependent system wherein each aspect of behavior can influence cognition through multiple processes. The intensity of activity performed likely moderates the PA and cognition relationship; however, current evidence is mixed, with some evidence suggesting that MVPA has a greater effect (72), whereas other studies indicate that LPA promotes better cognition (73). Recreational PA (including exercise training) might also be more beneficial to cognition than other types of PA, such as active transport or occupational activity. Cognitively passive and active SB (12) have distinct associations with cognition (65), such that cognitively passive SB is negatively associated with cognition and active SB is positively associated with cognition (74–76). Poor sleep quality, in addition to sleep quantity, is associated with a greater risk of MCI and dementia (77–79), and people with MCI exhibit less consistent sleep patterns than their non-MCI peers (21). These findings highlight the challenges of identifying how the 24-HAC interacts with cognition and suggest that it may be critical for future research to examine how each behavior is both independently *and* interdependently associated with cognition.

### Limitations and Future Studies

It is important to note some possible methodological limitations of the present study. We included a heterogeneous sample of older adults with MCI (ie, amnestic and nonamnestic, single- and multiple domain MCI), likely comprising individuals with slightly different pathologies and neuropsychological profiles. Our modest sample size may have prevented us from detecting more robust results when comparing cognition across different latent activity profiles. Even though each 24-HAC activity profile had an acceptable sample size, the most extreme profiles (“Physical Activity Masters” and “Sedentary Savants”) accounted for only 31.6% of our sample. We also did not explore potential differences (eg, stratified analysis) based on biological sex, race, and ethnicity. Although we observed differences in the proportion of females between the activity profiles, we did not further investigate it due to statistical power. Participants were recruited from British Columbia, Canada’s most physically active province, incurring unintentional selection bias reflected in our sample broadly meeting the current guidelines of PA. This also means that the included participants were likely more fit (eg, better cardiorespiratory function) than participants in previous studies.

Our analyses only investigated device-measured PA, SB, and sleep and did not account for wear-time on week and weekend days as inclusion criteria for data analysis. We also did not collect self-report measures of 24-HAC behaviors, which can provide valuable information about the context of these behaviors. We measured 24-HAC behaviors with wrist-worn accelerometry, which improves participant compliance and is in concert with most studies using accelerometry but is prone to PA overestimation and SB underestimation. We also did not account for napping in our estimates of sleep, which was indexed as SB. Finally, our study was cross-sectional; thus, we highlight the need for longitudinal studies to identify how 24-HAC composition may affect cognition. Future studies should: (1) harmonize approaches to capturing PA, SB, and sleep in older adults; (2) compare how other aspects of the 24-HAC beyond time use (ie, PA intensity, SB type, sleep quality) can affect cognition; (3) compare 24-HAC compositions between people with and without cognitive impairment; (4) investigate 24-HAC composition longitudinally to understand temporal effects and explore the predictive ability to discern distinct activity profiles based on the rate of conversion from MCI to AD, and (5) identify activity profiles not meeting the recommended amounts of 24-HAC behaviors at baseline and target tailored interventions for such profiles to maximize the efficacy of PA, SB, or sleep interventions on cognition.

## Conclusions

Older adults with MCI exhibited distinct 24-HAC activity profiles; however, cognition was similar across all 4 activity profiles. It is thus unclear whether 24-HAC composition is related to cognition in older adults with MCI. Future research must identify how qualitative aspects of the 24-HAC (eg, PA intensity or sleep quality) can influence cognition.

## Supplementary Material

glae099_suppl_Supplementary_Material

## Data Availability

Data will be made available on request.
