# Supplementary material for: The Association of the 24-Hour Activity Cycle Profiles With Cognition in Older Adults With Mild Cognitive Impairment: A Cross-Sectional Study
Source: J Gerontol A Biol Sci Med Sci. 2024 Apr 20;79(7):glae099. doi: 10.1093/gerona/glae099 (PMC11167489; doi:10.1093/gerona/glae099)
Supplement: glae099_suppl_Supplementary_Material [file glae099_suppl_supplementary_material.docx]

**SUPPLEMENTAL MATERIAL**

**Table of Contents**

[eTable 1. Detailed inclusion and exclusion criteria of the three randomized controlled trials included in the study. 2](#_Toc159941239)

[eTable 2. Fit statistics of the latent profile models with two to six profiles (n = 253). 6](#_Toc159941240)

[eTable 3. Demographic characteristics, time spent in 24-hour activity behaviours, and individual neuropsychological test scores as a function of randomized controlled trial. 7](#_Toc159941241)

[eTable 4. Fit statistics of the latent profile models with two to six profiles (n = 253) without outlier winsorization. 9](#_Toc159941242)

[eTable 5. Demographics, 24-hour activity cycle behaviours, and ADAS-Cog Plus by activity profile without outlier winsorization. 10](#_Toc159941243)

[eFigure 1. Flow chart of participants. 12](#_Toc159941244)

# eTable 1. Detailed inclusion and exclusion criteria of the three randomized controlled trials included in the study.

| **Studies** | **Inclusion criteria** | **Exclusion criteria** |
| --- | --- | --- |
| **Falck et al. (2020)** | Individuals were required to: 1) be aged 65–85 years; 2) live independently in their own homes; 3) score >24/30 on the MMSE; 4) score <26/30 on the MoCA; 5) have poor subjective sleep quality indicated by a score >5/21 on the Pittsburgh Sleep Quality Index (PSQI); 6) score <5/15 on the 15-item Geriatric Depression Scale; 7) be in sufﬁcient health to participate in regular physical activity as indicated by the Physical Activity Readiness Questionnaire; and 8) be able to understand, speak, and read English. | Excluded individuals who: 1) diagnosed with dementia of any type; 2) diagnosed with any progressive neurodegenerative disease (e.g., Parkinson’s disease); 3) diagnosed with obstructive sleep apnea; 4) receiving continuous positive air pressure treatment; 5) were at high risk for cardiac complications during PA; 6) had clinically important peripheral neuropathy that impacted mobility; or 7) severe musculoskeletal and/or joint disease which impaired mobility. |
| **Barha et al. (2022)** | Individuals were required to: 1) be aged 65–85 years; 2) have subjective memory complaints, defined as the self-reported feeling of worsening memory, as determined by a standard question; 3) score <26/30 on the MoCA; 4) score >22/30 on the MMSE and < 1.0 on the Clinical Dementia Rating; 5) do not have any significant impairment in daily function as indicated by a score of > 6/8 on the Lawton and Brody Instrumental Activities of Daily Living Scale; 6) score < 5/15 on the Geriatric Depression Scale; 7) completed high school education; 8) live independently in their own homes; 9) read, write, and speak English with acceptable visual and auditory acuity; 10) not expect to start, or be stable, on a fixed dose of anti-dementia medications (e.g., donepezil, galantamine); 11) provide a signed and dated informed consent document; 12) able to walk independently without an aid; 13) be in sufficient health to participate in the exercise programs, based on their medical history, the PAR-Q Plus, and written approval by their family physician (if required); and 14) have the capacity to comply with the scheduled assessments and exercise sessions. | Excluded individuals who: 1) engaged in moderate-intensity aerobic exercise > 60 minutes per week, in the 3 months prior to study entry; 2) engaged in progressive resistance training > 1×/week in the 3 months prior to study entry; 3) diagnosed previously with dementia of any type; 4) clinically suspected to have neurodegenerative disease as the cause of MCI that is not Alzheimer’s  disease, vascular cognitive impairment, or both (e.g., multiple sclerosis, Parkinson’s disease, Huntington’s disease, frontotemporal dementia); 5) at high risk for cardiac complications during exercise or unable to self-regulate activity or to understand recommended activity level; 6) diagnosed by their family physician with clinically important peripheral neuropathy or severe musculoskeletal or joint disease that impairs mobility; 7) on a new or recent (i.e., less than 3 months from study entry) or changed dose of medications that may negatively affect cognitive function, such as anticholinergics (i.e., typical and atypical antipsychotics) and anticonvulsants (e.g., gabapentin, valproic acid); 8) on any hormone therapy (estrogen, progesterone, or testosterone) in the last 24 months; or 9) planning to participate, or already enrolled in, a concurrent clinical drug or exercise trial. |
| **Liu-Ambrose et al. (2021)** | Individuals who fulfill the criteria for subcortical ischemic vascular cognitive impairment (SIVCI), defined as the presence of cognitive impairment combined with cerebral small vessel disease. Cognitive impairment was operationalized as a MoCA score < 26/30, and cerebral small vessel disease was defined as the presence of whit matter hyperintensities and/or lacunes on computed tomography (CT) or magnetic resonance imaging (MRI). Additional inclusion criteria required individuals to: 1) be aged 55 years or older; 2) MMSE score >20/30; 3) live independently in their own homes; 4) be able to comply with scheduled visits, treatment plan, and other trial procedures; 5) read, write, and speak English with acceptable visual and auditory acuity; 6) be on a fixed dose of cognitive medications that is not expected to change during the 12-month study period, or, if they are not on any of these medications, they are not expected to start them during the 12-month study period; 7) provide informed consent; 8) be able to walk independently; and 9) must be in sufficient health to participate in the progressive resistance training program. | Excluded individuals who: 1) had an absence of  cerebral small vessel disease on a brain CT or MRI; 2) were diagnosed with dementia of any type or another type of neurodegenerative or neurological condition (e.g., Parkinson’s disease); 3) were diagnosed with a genetic cause of SIVCI; 4) were at high risk for cardiac complications during exercise and/or unable to self-regulate or to understand the recommended activity level; 5) participated in regular resistance training in the last 6 months;  6) had clinically important peripheral neuropathy or severe musculoskeletal or joint disease that impairs mobility; 7) recently started taking medications (< 3 months prior to study) that may negatively affect cognitive function, such as anticholinergics; 8) were planning to participate, or already enrolled in, a clinical drug trial or exercise trial concurrent to this study; or 9) were unable to meet MRI scanning requirements, as specified by the UBC 3T MRI Research Center. |

# eTable 2. Fit statistics of the latent profile models with two to six profiles (n = 253).

| Classes | LogLik | AIC | BIC | CAIC | SABIC | ICL | Minimum N | BLRT (*p*-value) | Entropy |
| --- | --- | --- | --- | --- | --- | --- | --- | --- | --- |
| 2-class model | -136.07 | 298.14 | 344.07 | 357.07 | 302.86 | -378.87 | 79 | 0.010 | 0.80 |
| 3-class model | -86.49 | 212.98 | 283.65 | 303.65 | 220.24 | -325.00 | 40 | 0.010 | 0.84 |
| 4-class model | -76.55 | 207.1 | 302.5 | 329.5 | 216.9 | -393.93 | 25 | 0.049 | 0.75 |
| 5-class model | -69.55 | 207.09 | 327.23 | 361.23 | 219.44 | -441.59 | 26 | 0.337 | 0.73 |
| 6-class model | -50.92 | 183.84 | 328.7 | 369.7 | 198.73 | -440.77 | 7 | 0.029 | 0.76 |

# eTable 3. Demographic characteristics, time spent in 24-hour activity behaviours, and individual neuropsychological test scores as a function of randomized controlled trial.

| **Variables ^a^** | **Falck et al (2020)** | **Barha et al. (2022)** | **Liu-Ambrose et al. (2021)** | *p*-value |
| --- | --- | --- | --- | --- |
|  | (n = 88) | (n = 89) | (n = 76) |  |
|  | M (SD) | M (SD) | M (SD) |  |
| Demographics |  |  |  |  |
| Age, years | 73.58 (5.39) | 73.06 (5.15) | 74.55 (5.66) | 0.203 |
| Females, n (%) | 53 (60.2) | 56 (62.9) | 48 (63.2) | 0.908 |
| BMI, kg/m^2^ | 26.35 (4.20) | 25.82 (4.59) | 26.86 (4.97) | 0.345 |
| MoCA, score | 22.51 (2.39) | 21.87 (2.75) | 21.74 (3.37) | 0.165 |
| MMSE, score | 28.17 (1.39) | 27.53 (2.23) | 27.46 (1.94) | 0.026* |
| Education, n (%) |  |  |  | 0.343 |
| High school or less | 13 (14.7) | 11 (12.4) | 10 (13.2) |  |
| Some university | 26 (29.5) | 18 (20.2) | 14 (18.4) |  |
| Trade school | 11 (12.5) | 9 (10.1) | 5 ( 6.6) |  |
| University degree | 38 (43.2) | 51 (57.3) | 47 (61.8) |  |
| 24-hour activity cycle behaviours |  |  |  |  |
| Sleep, min/day | 434.63 (52.28) | 433.50 (47.65) | 417.47 (61.13) | 0.079 |
| Sleep, %/day | 30.18 (3.63) | 30.10 (3.31) | 28.99 (4.25) | 0.079 |
| Sedentary behaviour, min/day | 624.13 (122.53) | 634.51 (101.06) | 622.10 (132.92) | 0.765 |
| Sedentary behaviour, %/day | 43.34 (8.51) | 44.06 (7.02) | 43.20 (9.23) | 0.765 |
| Light PA, min/day | 282.37 (72.71) | 280.33 (63.81) | 299.21 (82.81) | 0.202 |
| Light PA, %/day | 19.61 (5.05) | 19.47 (4.43) | 20.78 (5.75) | 0.202 |
| Moderate-to-vigorous PA, min/day | 98.87 (62.78) | 91.65 (58.41) | 101.23 (76.83) | 0.616 |
| Moderate-to-vigorous PA, %/day | 6.87 (4.36) | 6.36 (4.06) | 7.03 (5.34) | 0.616 |
| Cognition |  |  |  |  |
| ADAS-Cog-Plus, score | -0.39 (0.55) | -0.34 (0.60) | -0.17 (0.71) | 0.066 |

Notes. ^a^ Data presented either as mean (standard deviation) or count (%) where applicable.

MoCA = Montreal Cognitive Assessment; MMSE = Mini-Mental State Examination; PA = physical activity; ADAS-Cog Plus = Alzheimer’s Disease Assessment Scale-Cognitive-Plus.

**p*<0.05.

# eTable 4. Fit statistics of the latent profile models with two to six profiles (n = 253) without outlier winsorization.

| Classes | LogLik | AIC | BIC | CAIC | SABIC | ICL | Minimum N | BLRT (*p*-value) | Entropy |
| --- | --- | --- | --- | --- | --- | --- | --- | --- | --- |
| 2-class model | -176.02 | 378.03 | 423.97 | 436.97 | 382.75 | -482.05 | 123 | 0.010 | 0.67 |
| 3-class model | -113.53 | 267.07 | 337.74 | 357.74 | 274.33 | -376.94 | 35 | 0.010 | 0.85 |
| 4-class model | -102.20 | 258.40 | 353.80 | 380.80 | 268.20 | -451.22 | 25 | 0.010 | 0.74 |
| 5-class model | -94.49 | 256.98 | 377.12 | 411.12 | 269.33 | -495.92 | 26 | 0.337 | 0.73 |
| 6-class model | -75.21 | 232.41 | 377.28 | 418.28 | 247.30 | -493.48 | 7 | 0.010 | 0.77 |

# eTable 5. Demographics, 24-hour activity cycle behaviours, and ADAS-Cog Plus by activity profile without outlier winsorization.

| **Variable** | **Overall**  **(n = 253)** | **Average**  **24-HAC**  **(n = 103)** | **Active Chillers**  **(n = 64)** | **Physical Activity Masters**  **(n = 54)** | **Sedentary Savants**  **(n = 26)** | ***p*** |
| --- | --- | --- | --- | --- | --- | --- |
|  | M (SD) | M (SD) | M (SD) | M (SD) | M (SD) |  |
| Demographics |  |  |  |  |  |  |
| Age, years | 73.69 (5.41) | 74.05 (5.21) | 74.09 (4.97) | 72.16 (5.37) | 74.52 (6.90) | 0.128 |
| Females, n (%) | 157 (62.10) | 71 (68.93) | 39 (55.71) | 40 (74.07) | 7 (26.92) | <0.001^a^ |
| BMI, kg/m^2^ | 26.32 (4.58) | 26.65 (4.69) | 26.23 (4.12) | 24.79 (4.10) | 28.42 (5.35) | 0.007^b^ |
| MoCA | 22.05 (2.85) | 22.13 (2.95) | 22.19 (2.92) | 21.94 (2.80) | 21.62 (2.37) | 0.824 |
| MMSE | 27.73 (1.90) | 27.97 (1.72) | 27.43 (2.32) | 27.74 (1.76) | 27.58 (1.58) | 0.313 |
| Day duration | 1344.83 (39.61) | 1342.55 (36.72) | 1350.94 (44.03) | 1334.07 (40.19) | 1359.75 (30.53) | 0.021^b^ |
| 24-HAC behaviours (%/day) |  |  |  |  |  |  |
| Sleep | 29.75 (3.98) | 29.69 (4.12) | 30.72 (3.76) | 28.69 (3.58) | 29.61 (4.35) | 0.043^c^ |
| Sedentary behaviour | 43.42 (8.66) | 42.63 (3.82) | 48.88 (3.62) | 31.32 (5.11) | 57.08 (2.26) | <0.001^d^ |
| Light PA | 19.85 (5.22) | 21.18 (2.91) | 16.45 (1.67) | 25.88 (3.76) | 11.17 (2.44) | <0.001^d^ |
| MVPA | 6.98 (5.32) | 6.50 (2.83) | 3.94 (2.02) | 14.12 (6.10) | 2.20 (1.47) | <0.001^e^ |
| Cognitive function |  |  |  |  |  |  |
| ADAS-Cog-Plus | -0.29 (0.66) | -0.24 (0.05)^f^ | -0.19 (0.06)^f^ | -0.31 (0.07)^f^ | -0.28 (0.10)^f^ | 0.602^f^ |

Notes: ^a^ *p*<.05 between “Average 24-HAC” vs. “Sedentary Savants,” “Physical Activity Masters” vs. “Sedentary Savants,” and “Active Chillers” vs. “Sedentary Savants.”

^b^ *p*<.05 between “Physical Activity Masters” vs. “Sedentary Savants.”

^c^ *p*<.05 between “Active Chillers” vs. “Physical Activity Masters.”

^d^ *p*<.05 between all the profiles.

^e^ *p*<.05 between all the profiles except between “Active Chillers” vs. “Sedentary Savants.”

^e^ Results presented as Analysis of Covariance (ANCOVA) model with data by profile presented as estimated marginal means and standard error, adjusted for biological sex, age, body mass index, Montreal Cognitive Assessment score, and RCT (*F* _(9, 243)_ = 25.75).

BMI = Body mass index; MoCA = Montreal Cognitive Assessment; MMSE = Mini-Mental State Examination; 24-HAC = 24-hour activity cycle; PA = physical activity; MVPA = moderate-to-vigorous physical activity; ADAS-Cog Plus = Alzheimer’s Disease Assessment Scale-Cognitive Plus. Data are presented either as mean (standard deviation) or count (%) where applicable. Results are presented for Analysis of Variance (ANOVA) models for continuous variables and chi-square test for categorical variables unless otherwise noted.


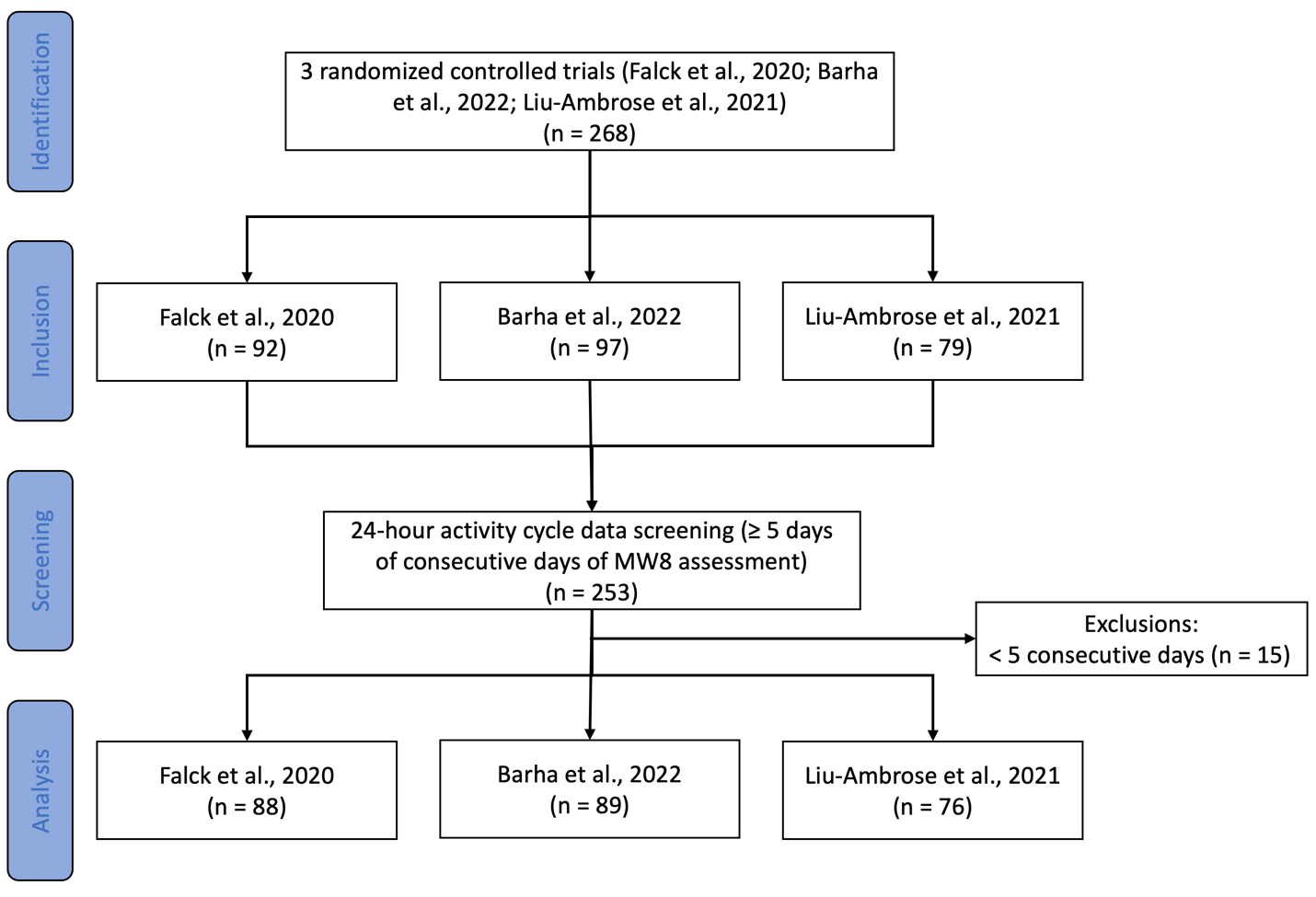


# eFigure 1. Flow chart of participants.
